# Why intrinsic reduced susceptibility in rare fungal pathogens should be differentiated from intrinsic resistance: an ISHAM conceptual framework and narrative review

**DOI:** 10.1128/aac.00263-26

**Published:** 2026-06-10

**Authors:** Cornelia Lass-Flörl, Stephan Steixner, Angelika Bauer, Jacques F. Meis, Arnaldo L. Colombo, Oliver A. Cornely, Arunaloke Chakrabarti, Ferry Hagen, Amanda Ribeiro dos Santos, Amir Seyedmousavi, Shivaprakash M. Rudramurthy, Roya Vahedi-Shahandashti, on behalf of ISHAM Working Group on Intrinsic Antifungal Resistance

**Affiliations:** 1 Institute of Hygiene and Medical Microbiology, ECMM Excellence Diamond Centre of Medical Mycology, Medical University of Innsbruck, Innsbruck, Austria; 2 Radboudumc-CWZ Center of Expertise for Mycology, Nijmegen, the Netherlands; 3 Institute of Translational Research, Cologne Excellence Cluster on Cellular Stress Responses in Aging-Associated Diseases (CECAD) and Excellence Center for Medical Mycology, University of Cologne, Cologne, Germany; 4 Antimicrobial Resistance Institute of São Paulo (ARIES), Federal University of São Paulo, São Paulo, Brazil; 5 Institute of Translational Research, Cologne Excellence Cluster on Cellular Stress Responses in Aging-Associated Diseases (CECAD), Faculty of Medicine, University of Cologne, Cologne, Germany; 6 Department I of Internal Medicine, Division of Infectious Diseases, Excellence Center for Medical Mycology (ECMM), Faculty of Medicine, University Hospital Cologne, Cologne, Germany; 7 German Centre for Infection Research (DZIF), Partner Site Bonn-Cologne, Cologne, Germany; 8 Faculty of Medicine and University Hospital Cologne, Clinical Trials Centre Cologne (ZKSKöln), University of Cologne 14309, Cologne, Germany; 9 Doodhadhari Burfani Hospital and Research Institute, Haridwar, India; 10 Department of Medical Microbiology, University Medical Center Utrecht, Utrecht, the Netherlands; 11 Westerdijk Fungal Biodiversity Institute 141042, Utrecht, the Netherlands; 12 Department of Clinical and Toxicological Analyses, School of Pharmaceutical Sciences, University of São Paulo, São Paulo, Brazil; 13 Microbiology Service, Department of Laboratory Medicine, Clinical Center, National Institutes of Health, Bethesda, Maryland, USA; 14 Department of Medical Microbiology, Post Graduate Institute of Medical Education and Research, , Chandigarh, India; Samsung Medical Center Department of Infectious Diseases, Seoul, Republic of Korea

**Keywords:** antifungal resistance, intrinsic resistance, acquired resistance, intrinsic reduced susceptibility, rare fungi

## Abstract

Accurate interpretation of antifungal minimum inhibitory concentrations (MICs) requires distinguishing species-level intrinsic phenotypes from isolate-level acquired resistance. Many fungi exhibit intrinsic reduced susceptibility (IRS) reflected by wild-type (WT) MIC distributions that are shifted toward higher values (uniformly or with a high-MIC tail). This may lead to the misclassification of high-end WT isolates as resistant and compromise surveillance and cross-center comparability, particularly for rare fungi lacking established breakpoints. In this review, we propose a pragmatic and standardized framework to distinguish intrinsic phenotypes (normal susceptibility, IRS, intrinsic resistance) from acquired resistance based on species-specific WT MIC distributions and standardized WT/non-wild-type (NWT) reporting. This framework is specifically intended to guide interpretation in data-limited settings where WT distributions and epidemiological cut-off values (ECOFFs) are not yet established. MICs within the unimodal species-specific WT range, even if high, should be interpreted as potentially reflecting IRS and not as acquired resistance. Only MICs clearly right-shifted beyond the WT range should be classified as NWT by epidemiological cut-off values or, if unavailable, as putative NWT, and considered candidates for acquired resistance. When breakpoints are lacking, WT versus NWT reporting with explicit comments on IRS/intrinsic resistance is preferable to forced susceptible/resistant (S/R) categorization. In the absence of established WT distributions or ECOFFs, such classification remains provisional and should rely on descriptive MIC patterns, available data sets, and clinical context. Integrating these biological interpretations with pharmacokinetics/pharmacodynamics and clinical context supports optimized dosing, avoids inappropriate drug exclusion, and enables consistent surveillance and multicenter comparisons.

## INTRODUCTION

Antifungal resistance is a growing challenge in clinical mycology, driven particularly by the limited number of available systemic antifungal drug classes and the growing burden of infections caused by opportunistic (1) and rare fungi (2). To interpret antifungal susceptibility testing results accurately and design meaningful surveillance studies, it is essential to distinguish between intrinsic resistance, intrinsic reduced susceptibility (IRS), and acquired resistance. It is also equally important to understand how these biological concepts relate to MIC distributions and interpretative frameworks, including epidemiological cut-off values (ECOFF; wild type [WT] versus non-wild type [NWT]) as well as the susceptibility categories defined by the Clinical and Laboratory Standards Institute (CLSI) and the European Committee on Antimicrobial Susceptibility Testing (EUCAST), particularly when clinical breakpoints are lacking. Despite their clear biological distinctions, IRS is frequently conflated in the literature with intrinsic (innate) resistance, creating conceptual ambiguity and complicating the interpretation of susceptibility data. In this narrative review, we emphasize the need to define IRS as a distinct species-level phenotype, particularly for rare fungi where interpretive criteria are limited, and this differentiation has important implications for surveillance, reporting, and therapy. Developed within the ISHAM Working Group on Intrinsic Resistance, this framework aims to clarify the distinctions between intrinsic resistance, IRS, and acquired resistance in rare fungal pathogens.

## PHENOTYPIC CATEGORIES VERSUS BIOLOGICAL CONCEPTS

EUCAST and CLSI provide clinical breakpoints to guide treatment decisions for individual isolates, classifying results into categories, such as S (susceptible), I/SDD (susceptible with increased exposure or dose-dependent), and R (resistant) according to EUCAST (3–5) and S, intermediate or SDD (susceptible, dose-dependent), and R according to CLSI (6, 7). These categories represent strictly isolate-level interpretations based on measured MICs and species-specific breakpoints. In parallel, both EUCAST and CLSI also refer to species-based concepts: CLSI formally uses the term intrinsic resistance, while EUCAST has adopted the terms ‘expected resistant phenotype’ and ‘expected susceptible phenotype’ for species–drug combinations in which susceptibility or resistance can be inferred from species identification alone (8, 9). However, these species-level concepts are not consistently or mechanistically defined, and their boundaries relative to isolate-level interpretations remain unclear. In particular, IRS is largely neglected as a distinct biological phenotype and often subsumed under intrinsic resistance despite reflecting a fundamentally different species-level MIC distribution. To avoid conceptual ambiguity, it is essential to clearly separate species-level biological properties, intrinsic resistance and IRS, from isolate-level phenomena, such as acquired resistance and clinical categorization (S/I/SDD/R), which arise from specific resistance mechanisms in individual strains (3–5). In this review, the terms intrinsic resistance and innate resistance are used synonymously to describe species-level resistance arising from inherent genetic properties. The term “expected resistant phenotype,” as used by EUCAST, refers to the same biological concept but is expressed within a clinical interpretive framework. To avoid ambiguity, we use “intrinsic resistance” as the primary term throughout this manuscript when referring to this species-level property. In contrast, IRS represents a distinct species-level phenotype and should not be conflated with these terms.

### Different resistance type definitions

#### Intrinsic resistance

Intrinsic resistance is a species- or species-complex-level trait (10) that arises from the organism’s inherent genetic makeup and expressed uniformly across its WT population. A species is considered intrinsically resistant to a drug or drug class when all or the vast majority of its WT isolates exhibit MICs so high that clinical efficacy is not expected, regardless of prior antifungal exposure (3–5). According to the CLSI M100 document, a ≥97% rule (3% cutoff) is used to define intrinsic resistance. In other words, if 97% or more isolates of a species fail to be inhibited at or near the highest test concentration, the species is classified as intrinsically resistant (8). Under these circumstances, susceptibility testing offers little clinical value because resistance is essentially universal. However, the ≥97% criterion should be interpreted with caution, particularly for rare fungal species and data sets with limited isolate numbers, where the observed proportion of resistant isolates may be influenced by sampling bias and may not reflect the true species-level MIC distribution. The application of a fixed percentage threshold is further complicated when MIC distributions are multimodal or broadened, as such patterns indicate population heterogeneity rather than a uniform species-wide phenotype. For example, in outbreak settings, such as *Candida auris*, a high proportion of isolates may reflect the dominance of a single clonal lineage rather than a uniform species-wide phenotype. In practical terms, intrinsic resistance should not be assigned based on proportion alone. Instead, classification requires evidence of a consistently elevated MIC distribution across genetically diverse isolates, supported by analysis of distribution shape and, where available, comparison with external or multicenter data sets. In clonal outbreak settings, high resistance rates should be interpreted cautiously and not assumed to represent the underlying species biology.

These considerations highlight important limitations of applying a fixed proportion-based definition of intrinsic resistance, particularly in the context of rare fungi, heterogeneous MIC distributions, and outbreak-driven population structures. Accordingly, no universal MIC threshold can be defined to distinguish intrinsic resistance from IRS across species and antifungal agents. This distinction must instead be based on the overall MIC distribution, its relationship to achievable drug exposure pharmacokinetic/pharmacodynamic (PK/PD), and available clinical evidence.

#### Intrinsic reduced susceptibility (IRS)

IRS refers to a species-level phenotype in which the WT MIC distribution is shifted toward higher values compared with an expected WT susceptibility range based on available data, yet does not meet the ≥97% criterion required to classify the species as intrinsically resistant. This highlights a limitation of proportion-based definitions, as species with elevated but non-uniform MIC distributions may be misclassified if distributional structure and population heterogeneity are not considered. Unlike intrinsic resistance, which is uniform, IRS represents an inherent but graded reduction in susceptibility, where the WT population may remain treatable, particularly with optimized or increased drug exposure.

In practice, IRS should be inferred from the position and shape of the WT MIC distribution relative to the expected WT susceptibility range for the species–drug combination. It is characterized by a consistent rightward shift across multiple twofold dilutions without evidence of a distinct non-WT subpopulation. In the absence of established WT distributions or ECOFFs, which is common for rare fungi, formal WT/NWT classification is not possible. In such settings, IRS should be inferred from MIC distribution patterns, reproducibility across data sets, and consistency with PK/PD constraints and clinical evidence, and any classification should be considered provisional. No universal MIC threshold can reliably distinguish intrinsic resistance from IRS. Instead, this distinction should be based on whether the WT MIC distribution lies within, or beyond, the range compatible with clinically achievable drug exposure, integrating PK/PD considerations and available clinical evidence.

For rare fungi, this approach is inherently more challenging due to limited isolate numbers and the difficulty of excluding acquired resistance mechanisms. In such settings, WT populations are inferred from MIC data under the assumption that most isolates lack acquired resistance, consistent with EUCAST and CLSI principles, and routine sequencing of all isolates is not required. However, molecular analyses may be applied selectively to investigate isolates with atypical MIC values or suspected resistance. Consequently, ECOFFs and IRS classification in rare species should be interpreted with caution and, where possible, supported by multicenter data sets, PK/PD considerations, and accumulating clinical experience.

IRS category can include two biological scenarios.

##### Homogeneous WT shift driven by shared genetic determinants (IRS type A)

The first scenario represents a broad and homogeneous shift of the WT MIC distribution that arises from a genetic characteristic shared by nearly all members of a species. In this pattern, MIC values are consistently elevated across the entire WT population not because isolates have acquired resistance mechanisms, but because the species inherently carries fixed polymorphisms that reduce susceptibility. In practice, IRS type A may be identified by a unimodal and relatively narrow WT MIC distribution that is consistently shifted as a whole compared with the expected WT susceptibility range without evidence of distributional broadening or a distinct high-MIC subpopulation. A classic example is *Candida parapsilosis* and echinocandins: all isolates possess the naturally occurring Fks1 P660A polymorphism, which uniformly increases echinocandin MICs across the species (11–14). Although this elevation does not render the drug class entirely ineffective, it does shift the MIC distribution upward in a predictable manner. This inherent, species-wide shift was the rationale for introducing species-specific EUCAST breakpoints (4), ensuring that WT isolates are not incorrectly categorized as resistant. This scenario exemplifies IRS as a uniform biological trait of the species rather than an adaptive response arising during antifungal exposure.

##### Heterogeneous WT distribution with a high-MIC tail (IRS type B)

The second scenario involves a heterogeneous WT population with a high-MIC tail. In this pattern, most isolates display moderately elevated MICs, but a substantial minority exhibits markedly high MICs, even in the absence of prior antifungal exposure. These high MICs reflect natural population heterogeneity rather than acquired resistance. Operationally, IRS type B is characterized by a broader WT MIC distribution in which most isolates cluster around a central mode, but a reproducible upper tail of higher MIC values is present without clear separation from the main population. This pattern reflects intrinsic population heterogeneity rather than the emergence of a distinct non-WT population. This pattern provides a biological framework for interpreting multimodal or broadened MIC distributions, which may otherwise be misclassified as intrinsic resistance when assessed using proportion-based criteria alone. *Nakaseomyces glabratus* (formerly *Candida glabrata*) is the classic example: its WT population spans a wide MIC range, with roughly 10–30% of isolates exhibiting high fluconazole MICs (>32 mg/L) despite no prior exposure (15–19). A similar situation has been noted for *Candida auris*, mainly Clade I, which was initially proposed to be intrinsically resistant to fluconazole (20–22). However, this classification does not meet the ≥97% criterion for intrinsic resistance. Although more than 93% of tested isolates exhibit high fluconazole MICs (23), this observation is likely influenced by clonal expansion during hospital outbreaks, most prominently involving clades I, III, and IV, where many analyzed isolates are genetically identical or closely related (24, 25). As a result, the apparent prevalence of fluconazole resistance in *C. auris* is strongly shaped by clade distribution (26), geographic region (25), and the duration of endemic circulation within individual hospitals (27), rather than reflecting a uniform species-wide phenotype (28). This example illustrates how the ≥97% rule may be confounded by clonal expansion and geographic clustering, reinforcing the need to interpret proportion-based criteria within the context of population structure and epidemiology.

In the absence of established ECOFFs, identification of non-WT isolates should rely on detection of a clear separation from the main WT distribution rather than applying arbitrary dilution thresholds. In IRS type B distributions, elevated MIC values may still fall within the natural WT range and should, therefore, not be interpreted as acquired resistance without supporting genetic or epidemiological evidence. This issue is particularly relevant for rare filamentous fungi, including *Fusarium* spp. (29, 30), *Scedosporium*/*Lomentospora* (31), *Paecilomyces* (32, 33), *Scopulariopsis* (34, 35), and dematiaceous (black yeast-like) fungi (36, 37), where MIC distributions are often heterogeneous, and ECOFFs are lacking; in such cases, classification into IRS type A or B should be considered provisional and inferred from distributional patterns and consistency with biological and PK/PD considerations.

For laboratories with limited isolate numbers, interpretation should be guided by comparison with published or multicenter data sets, as small sample sizes may not reliably capture distribution shape (e.g., the presence of a high-MIC tail) and may lead to misclassification. Where robust WT distributions cannot be defined, classification should remain cautious and provisional.

### Acquired resistance

Acquired resistance occurs when only a subset of isolates within a species develops elevated MICs due to specific genetic changes, often under antifungal exposure (3–6, 13, 15). In this scenario, the baseline WT population of the species remains susceptible or displays IRS, while only a fraction of isolates acquires additional genetic alterations and becomes NWT, with MICs rising above the ECOFF. Acquired resistance is, therefore, an isolate-level phenomenon, even though resistant subpopulations may become frequent in certain species, regions, or patient groups. Moreover, multiple acquired resistance mechanisms may exist within a given species-antifungal combination.

A well-known example is azole-resistant *Aspergillus fumigatus*, in which mutations in *cyp51A* (e.g., TR₃₄/L98H and TR₄₆/Y121F/T289A) confer high MICs to multiple triazoles. These mutations may develop during prolonged azole therapy or arise from environmental exposure to agricultural azoles (4, 5). The species as a whole remains intrinsically susceptible, but mutant isolates represent acquired-resistant, acquired NWT subpopulations.

A second key example is echinocandin-resistant *Candida* spp., where resistance is strongly linked to *FKS1*/*FKS2* hotspot mutations. In species such as *Candida albicans*, *C. auris,* and *N. glabratus*, resistant isolates display MICs well above the WT distribution and above species-specific R breakpoints. They are clearly classified as resistant by EUCAST and CLSI (3, 6, 14, 15). Similar acquired mechanisms can affect azoles (e.g., *ERG11* mutations and efflux pump upregulation), again shifting MICs above the WT range and ECOFF (4, 12, 13). Although the definition of acquired resistance is generic (NWT MICs above the ECOFF), its frequency, mechanisms, and clinical impact are highly species- and drug-dependent.

**Fig 1:**
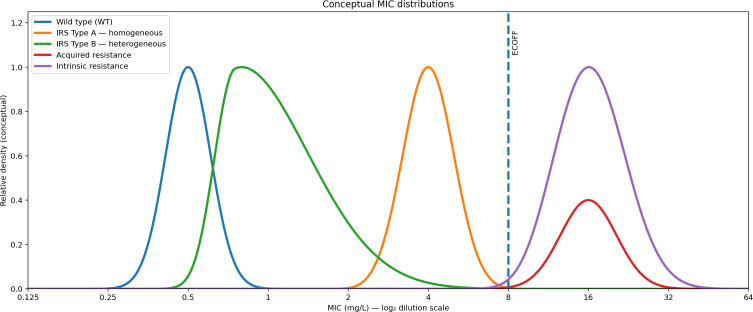
Conceptual MIC distributions. Schematic MIC distributions illustrating wild-type (WT), intrinsic reduced susceptibility (IRS), intrinsic resistance, and acquired resistance. The WT population is centered at low MIC values, whereas IRS type A shows a uniform species-level shift of the WT distribution, and IRS type B displays a broader WT distribution with a rightward tail. Acquired resistance is represented by a subset of isolates with elevated MICs relative to the WT population, while intrinsic resistance is characterized by uniformly high MICs across the species. The dashed line indicates the epidemiological cut-off value (ECOFF). IRS: intrinsic reduced susceptibility.

Importantly, acquired resistance can appear on top of an IRS background. For instance, in *C. parapsilosis*, *FKS1* mutations can occur in addition to the natural P660A polymorphism, moving MICs from an intrinsically elevated but still treatable WT range into a frankly resistant range; these isolates are NWT and often associated with clinical failure (14, 15). In such cases, it is helpful to distinguish WT IRS isolates (high but WT MICs) from acquired-resistant isolates arising within an IRS species. Fig. 1 provides a schematic overview of MIC distributions for WT, IRS, and different resistance phenotypes, and Table 1 summarizes the conceptual distinctions between intrinsic resistance, IRS, acquired resistance, and EUCAST/CLSI interpretive categories.

**TABLE 1:** Conceptual differences between intrinsic resistance, IRS, acquired resistance, and EUCAST/CLSI categories^*a*^

Term	Biological level	Primary determinant	MIC patterns	Definition/biological meaning	Clinical interpretation	Example species/drug	Key pitfalls
Intrinsic resistance	Species/species complex	WT MIC distribution	Uniformly elevated WT MICs (highly relative to achievable drug exposure)	Species-level characteristic in which all isolates are inherently not susceptible to a drug at clinically achievable exposure levels, independent of prior exposure	Drug considered ineffective	*P. kudriavzevii* vs. fluconazole; *L. prolificans* vs. all available antifungals, except olorofim, manogepix; *Trichosporonaceae* vs. echinocandins; *Cryptococcu*s vs. echinocandins	Not defined by proportion of isolates with high MICs; reflects a uniform species-level property
IRS type A (homogeneous)	Species/species complex	WT MIC distribution	Uniformly shifted WT MIC distribution, remaining narrow and unimodal, relative to the expected WT susceptibility range for the species–drug combination, remaining within the WT range (≤ ECOFF), where defined	Species-level phenotype characterized by a uniform shift toward higher MICs due to shared intrinsic genetic traits across isolates, relative to more susceptible species	Therapeutic window may persist with optimized exposure	*C. parapsilosis* vs. echinocandins	Not an isolate-level classification; elevated WT MICs should not be interpreted as acquired resistance; species-specific ECOFFs are often required
IRS type B (heterogeneous)	Species/species complex	WT MIC distribution with a right-shifted high-MIC tail	Broad WT MIC distribution with a right-shifted high-MIC tail, relative to the expected WT susceptibility range for the species–drug combination, remaining within the WT range (≤ ECOFF), where defined	Species-level phenotype characterized by natural heterogeneity in susceptibility, with a subset of WT isolates exhibiting higher MICs in the absence of acquired resistance mechanisms	Treatment outcome uncertain; caution advised	*N. glabratus* (*C. glabrata*) vs. fluconazole; *Candida auris* vs. fluconazole; *P. kudriavzevii* (*C. krusei*) vs. amphotericin B; *A. terreus* vs. amphotericin B	Not an isolate-level classification; high-MIC WT isolates may be misclassified as resistant; reflects natural population heterogeneity rather than acquired resistance
Acquired resistance	Isolate (subpopulation within a predominantly WT population)	ECOFF	Distinct NWT MIC distribution > ECOFF	Isolate-level phenomenon in which specific genetic alterations (e.g., *cyp51A, FKS, ERG11*, and efflux) result in reduced susceptibility compared with the WT population	Treatment likely ineffective; alternative therapy advised	*cyp51A* TR₃₄/L98H or TR₄₆/Y121F/T289A in *A. fumigatus* (azole resistance); *FKS1/FKS2* mutations in *Candida* spp. (echinocandin resistance)	Defined by deviation from the WT distribution (NWT), not by S/I/SDD/R categories alone; NWT isolates often, but not always, fall above clinical R breakpoints; may occur on top of an IRS background
WT	Species/species complex and isolate	ECOFF	Unimodal MIC distribution within the WT range (≤ ECOFF)	Population of isolates without phenotypically detectable acquired resistance mechanisms	Does not imply clinical susceptibility; treatment suitability depends on species-level susceptibility pattern (normal, IRS, or intrinsic resistance) and PK/PD considerations	WT distributions of *C. albicans* vs. echinocandins or triazoles; WT isolates of *L*. *prolificans* (intrinsically resistant to most currently available antifungals and, therefore, not clinically treatable)	WT ≠ susceptible; WT isolates may exhibit IRS or intrinsic resistance; clinical interpretation depends on species, and PK/PD
NWT	Species/species complex and isolate	ECOFF	MIC above the WT range (>ECOFF)	Isolate with MIC values exceeding the WT distribution, indicating reduced susceptibility	May indicate reduced likelihood of treatment success; treatment may be compromised	*FKS*-mutant *N. glabratus* with echinocandin MICs above the ECOFF	NWT is a biological concept, not a clinical category; NWT isolates often, but not always, fall into the clinical R category, where breakpoints exist
EUCAST S	Isolate	CBP	MICs ≤ S breakpoint according to EUCAST	Classification indicating a high probability of therapeutic success with standard dosing regimens	Treatment recommended	WT *C. albicans* with very low echinocandin MICs	S is a clinical interpretation, not a synonym with WT; some WT isolates in IRS species may be classified as I/SDD or even IE rather than S
EUCAST I	Isolate	CBP	MICs in the I range according to EUCAST	Classification indicating a high probability of therapeutic success when exposure is increased (e.g., higher dose, altered dosing regimen, or optimized drug delivery)	Treatment possible with optimized dosing	*C. parapsilosis* WT isolate with a relatively high echinocandin MIC that falls in the I range	I (like CLSI SDD) is a clinical label, not a biological concept; IRS species often yield WT isolates classified as I
EUCAST R	Isolate	CBP	MICs > R breakpoint according to EUCAST	Classification indicating a low probability of therapeutic success, even with increased exposure	Treatment not recommended, even with high exposure	*FKS*-mutant *N. glabratus* isolates with echinocandin MICs exceeding EUCAST R breakpoints	R is clinically defined (based on PK/PD and outcome) and not identical to NWT, although most R isolates are NWT
CLSI SDD	Isolate	CBP	MICs within the SDD range according to CLSI	Clinical analog to EUCAST I; classification indicating that therapeutic success is likely if higher or optimized dosing is used	Treatment possible with higher or optimized dosing	*N. glabratus* with intermediate fluconazole MICs interpreted as SDD in CLSI	SDD ≠ IRS; SDD is an isolate-level clinical category, whereas IRS is a species-level biological property
CLSI S/R	Isolate	CBP	MIC ≤ S breakpoint/MICs > R breakpoint, according to CLSI	Classification indicating high likelihood of therapeutic success (S) or failure (R) based on achievable drug exposure	S: treatment recommended; R: treatment not recommended (likely failure, even with high dosing)		Applicable only where CLSI breakpoints exist; S/R are clinical categories and not equivalent to WT/NWT

^*a*^ WT, wild type; NWT, non–wild type; MIC, minimum inhibitory concentration; ECOFF, epidemiological cutoff; CBP, clinical breakpoint; IRS, intrinsic reduced susceptibility; EUCAST, European Committee on Antimicrobial Susceptibility Testing; R, resistant; S, susceptible; I, susceptible, increased exposure; IE, insufficient evidence; CLSI, Clinical and Laboratory Standards Institute; SDD, susceptible, dose-dependent; PK/PD, stands for Pharmacokinetics/Pharmacodynamics.

## CLINICAL IMPLICATIONS OF INTRINSIC, IRS, AND ACQUIRED RESISTANCE

Distinguishing intrinsic resistance, IRS, and acquired resistance is essential for the appropriate use and interpretation of antifungal susceptibility testing. In cases of intrinsic resistance for specific fungal species, MIC testing has little clinical value, as uniformly high MICs are predictable at the species level and should prompt selection of alternative antifungal classes (8). In contrast, IRS represents an innate species-level shift in MIC distributions in which susceptibility testing remains clinically relevant but must be interpreted using species-specific breakpoints or ECOFFs to avoid misclassification of WT isolates as resistant. Acquired resistance, by comparison, is an isolate-level phenomenon in which MIC testing is critical (38), as detection of resistant isolates with elevated MICs directly informs therapeutic decisions, predicts treatment failure, and supports surveillance and infection control. Failure to distinguish these resistance categories may lead to unnecessary testing, misinterpretation of MIC data, inappropriate antifungal selection, or delayed initiation of effective therapy. At the same time, WT/NWT classification should not be interpreted as a direct proxy for clinical efficacy. While it provides a microbiological distinction between isolates with and without acquired resistance mechanisms, it does not, on its own, indicate whether a given antifungal agent is likely to be effective in a specific clinical setting. Interpretation of WT MICs, particularly in species with IRS, requires integration of PK/PD considerations, achievable drug exposure, and clinical context.

## MIC DISTRIBUTIONS, ECOFFs, AND THE ROLE OF EUCAST I AND CLSI SDD

In practice, the relationship between IRS and the clinical interpretation categories used by EUCAST and CLSI can be confusing. Category I by EUCAST (susceptible, increased exposure) and CLSI I/SDD (Intermediate or susceptible-dose-dependent) are isolate-level clinical interpretations indicating whether a specific isolate is likely to respond to standard or optimized dosing. In contrast, IRS is a species-level biological descriptor that reflects the position and shape of the WT MIC distribution (3–7). These concepts must, therefore, be kept conceptually distinct.

Importantly, MIC distributions and the resulting ECOFF values are method-dependent. Differences between antifungal susceptibility testing methodologies, including broth microdilution (EUCAST vs. CLSI), gradient diffusion methods, incubation time, and endpoint reading criteria, may lead to systematic shifts in MIC values and distribution shapes (14, 38–40). Consequently, ECOFFs are specific to the testing method used and should not be directly transferred across methodologies. When interpreting MIC distributions for the purpose of defining IRS or intrinsic resistance, results should, therefore, be compared only within the same methodological framework, and caution is required when integrating data from different testing platforms.

EUCAST and CLSI assign S, I/SDD, or R categories only when clinical breakpoints are available. These categories indicate whether a given isolate, with its specific MIC, is expected to respond to standard dosing (S), requires optimized or increased exposure (I/SDD), or is unlikely to respond, even with high exposure (R) (3–7). These categories describe expected treatment response for an individual isolate but do not define whether a species is intrinsically susceptible, displays IRS, or is intrinsically resistant. Such species-level characteristics cannot be determined from categorical interpretations (S/I/SDD/R) alone, but require evaluation of MIC distributions, ECOFFs, and their relationship to PK/PD parameters (3–5, 41).

In a fully susceptible species, such as *C. albicans* tested with an echinocandin, the WT MICs cluster at very low values, and isolates carrying *FKS* mutations form a clearly separated NWT tail above the ECOFF and above the clinical R breakpoint (3–5, 41). By contrast, in an IRS species, such as *C. parapsilosis* with echinocandins, the entire WT MIC distribution is shifted toward higher values compared to other *Candida* species. EUCAST and CLSI compensate for this by using species-specific breakpoints (3, 4, 42). A WT isolate of *C. parapsilosis* may, therefore, be categorized as S or I/SDD, even though its MIC would be considered high if interpreted with *C. albicans* breakpoints. Only isolates with MICs exceeding the WT distribution of *C. parapsilosis* (i.e., above the ECOFF) are interpreted as NWT and are candidates for acquired resistance; where species-specific clinical breakpoints exist, such isolates will often, but not always, fall into the R category.

By definition, IRS describes the WT MIC distribution itself and does not include resistant NWT outliers. WT isolates of an IRS species exhibit MICs that are elevated relative to the expected WT susceptibility range based on available data for the species–drug combination; however, as long as they remain within the WT range (≤ECOFF), they are not considered resistant and are interpreted as S, I/SDD, or insufficient evidence (IE) depending on species-specific breakpoints, PK/PD considerations, and guideline recommendations (3–7, 41, 42). Only isolates with MICs clearly above the ECOFF are classified as NWT and strongly suspected to have acquired resistance, even if they originate from an IRS species; whether they are categorized as clinically “R” depends on the availability and position of species-specific breakpoints.

In some species–drug combinations, such as *N. glabratus* and fluconazole, EUCAST considers the pair a poor target and labels it IE or refrains from providing S/I/R breakpoints at all (4, 5). CLSI likewise may only provide limited interpretive categories (e.g., SDD/R without S) or no breakpoints for uncommon species (6, 7, 43). These decisions reflect IRS and unfavorable PK/PD, but the term IRS itself is not used as a formal category in EUCAST or CLSI tables. For the purposes of rare fungus surveillance and ISHAM Working Group activities, it is, therefore, helpful to explicitly state IRS at the species level and then, within that framework, to classify individual isolates as WT versus NWT (and S/I/SDD/R where possible) according to ECOFFs and available breakpoints (3–5, 7, 41, 43).

## DISTINGUISHING IRS FROM INTRINSIC RESISTANCE WHEN NO BREAKPOINTS EXIST

In medical mycology, rare molds, some of which are considered emerging pathogens, are defined as pathogenic filamentous fungi other than *Aspergillus* species and members of the order *Mucorales* (44, 45). However, this definition is pragmatic, and certain uncommon or cryptic species may also be considered “rare” in a clinical context. Although these latter groups remain the most common causes of invasive mold infections, rare molds constitute a diverse and increasingly recognized group of opportunistic pathogens, particularly among immunocompromised patients. They encompass a broad range of genera, including, but not limited to *Fusarium*, *Lomentospora*, *Scedosporium*, *Paecilomyces*, *Scopulariopsis*, *Cladophialophora*, *Exophiala*, and *Alternaria*. Many of these organisms exhibit intrinsic resistance or reduced susceptibility to conventional antifungal agents. Importantly, the designation “rare” is context-dependent: species frequency varies markedly across geographic regions, climates, and patient populations, such that a mold considered rare in one setting may be encountered more frequently in another. Consequently, characterization of species-specific MIC distributions within distinct geographical contexts is essential, as local epidemiology, an aspect of “epidemiological geography,” strongly influences baseline susceptibility patterns, which must be interpreted within a consistent clinical framework. These diagnostic and therapeutic challenges have been comprehensively addressed elsewhere, which provides a framework for improved diagnosis and clinical management (44, 45).

For rare fungi, ECOFFs have not yet been established for most species–antifungal combinations. In such cases, a pragmatic approach consistent with EUCAST methodology is to use available data (including multicenter collections) to reconstruct approximate MIC distributions and define a modal MIC (or narrow mode) as the center of the putative WT peak (3–5). Isolates with MICs clearly above this range (e.g., more than two log dilutions) can reasonably be flagged as isolates with decreased susceptibility and suspected acquired resistance; strictly speaking, these should be regarded as putative NWT until a formal ECOFF has been established (4, 41). Importantly, this approach assumes that the majority of isolates represent the WT population, although the presence of undetected acquired resistance mechanisms cannot be fully excluded, particularly in small or geographically restricted data sets. Routine sequencing of all isolates is not required for WT definition; however, molecular analysis may be applied selectively to investigate outliers or to confirm suspected resistance mechanisms.

Where available, ECOFFs allow a crucial distinction. In their absence, classification must rely on approximate WT ranges and should be considered provisional. Isolates with MICs at or below the ECOFF are considered WT for that species–drug pair. Isolates with MICs above the ECOFF are NWT and likely harbor acquired resistance mechanisms, even if the clinical impact is not fully defined (4, 7, 41).

In this framework, IRS refers to the position of the WT MIC distribution itself. A species with IRS simply has a WT distribution that is located at higher MIC values compared with the expected WT susceptibility range based on available data for the species–drug combination. By contrast, isolates with MICs clearly above the species’ WT range (i.e., above the ECOFF) should be regarded as NWT, indicating additional acquired resistance mechanisms on top of the intrinsic background and no longer belonging to the IRS WT population.

At the same time, PK/PD and available clinical data can be used for assessing whether the WT MIC range is compatible with effective drug exposure. When the WT MIC distribution lies largely or entirely above concentrations that can be safely achieved in patients, the species–drug combination can be regarded as intrinsically resistant in practical terms, even in the absence of formally defined clinical breakpoints or ECOFFs (3–5, 46–48). This situation is exemplified by *L. prolificans*, which exhibits uniformly high MICs to most currently available antifungal agents, and by *Scedosporium* spp. tested against amphotericin B, for which achievable exposures are insufficient to overcome the inherent lack of activity (46–48).

## PRACTICAL IMPLICATIONS FOR RARE FUNGAL PATHOGENS

As WT/NWT classification alone does not directly inform treatment decisions, its clinical utility depends on appropriate contextual interpretation integrating PK/PD and species-specific knowledge. For a registry or working group focusing on intrinsic resistance in rare fungi, the following practical interpretive framework is useful.

### Define species-level susceptibility patterns

For each species–drug combination, baseline behavior should be assessed at the species level using WT MIC distributions, available PK/PD data, and published clinical outcomes to determine whether the pattern is best described as normal susceptibility, IRS, or intrinsic resistance (3–5, 17, 41, 42, 46–49).

### Use WT MIC distributions to distinguish IRS from acquired resistance

In IRS species, WT MIC distributions are shifted toward higher values either as a uniform shift (IRS type A) or as a broader distribution with a high-MIC tail (IRS type B). MICs that remain within the species’ WT distribution, even if high, should be interpreted IRS and not as acquired resistance. Only MICs clearly right-shifted beyond the species-specific WT range should be classified as NWT using an ECOFF where available or as putative NWT in species without a formal ECOFF and considered candidates for acquired resistance. Absolute MIC values should not be compared across species to infer resistance when WT ranges differ (4, 7, 41, 43).

### Do not force S/R categorization when breakpoints are lacking

In the absence of clinical breakpoints, especially for rare fungi, reporting WT versus NWT status, accompanied by an explicit comment on intrinsic resistance or IRS, is more scientifically appropriate than assigning arbitrary susceptible or resistant labels. However, to support clinical decision-making, such reports should ideally be accompanied by an interpretive comment that places the result into context, for example by indicating whether the species–drug combination is generally considered a reasonable therapeutic option, requires increased exposure, or is unlikely to be effective based on current PK/PD and clinical knowledge.

### Integrate biology with clinical decision-making while keeping layers distinct

Therapeutic decisions should integrate species-level susceptibility patterns, isolate-level MIC/NWT status, PK/PD considerations, and achievable drug exposures to judge whether a species–drug combination is a viable target, requires optimized dosing, or should be avoided. Maintain a clear separation between biological categories (intrinsic resistance, IRS, acquired resistance) and clinical interpretive categories (S, I/SDD, R) to support accurate surveillance, consistent reporting, and meaningful multicenter comparisons.

## CONCLUSION

When interpreting antifungal susceptibility data in clinically relevant fungi, it is crucial to distinguish between intrinsic resistance, IRS, and acquired resistance and not to mix these biological concepts with the clinical categories S, I/SDD, and R used by EUCAST and CLSI. Intrinsic resistance is a species-level trait in which the WT MICs are so high that a drug class is essentially ineffective. IRS is also a species-level feature but represents a less marked reduction in susceptibility. The WT MIC distribution is shifted toward higher values compared with the expected WT susceptibility range for the species–drug combination, yet a therapeutic window may still exist. Acquired resistance, by contrast, is an isolate-level phenomenon in which individual strains develop MICs above the WT range (above the ECOFF) typically driven by defined resistance mechanisms.

EUCAST and CLSI categories (S, I/SDD, R, IE) apply only when breakpoints exist and inform about treatment decisions for individual isolates; they do not define whether a species exhibits IRS or intrinsic resistance. For many rare fungi, where breakpoints are lacking, interpretation must, therefore rely on MIC distributions, WT versus NWT classification, and PK/PD considerations. Importantly, IRS itself is not uniform and may manifest either as a homogeneous, species-wide WT shift or as a heterogeneous WT distribution with a high-MIC tail. Explicitly recognizing these distinct IRS patterns helps to separate innate species biology from true acquired resistance and improves the consistency of surveillance, reporting, and clinical interpretation.
